# Next-generation tissue microarray (ngTMA) increases the quality of biomarker studies: an example using CD3, CD8, and CD45RO in the tumor microenvironment of six different solid tumor types

**DOI:** 10.1186/1479-5876-11-104

**Published:** 2013-04-30

**Authors:** Inti Zlobec, Viktor H Koelzer, Heather Dawson, Aurel Perren, Alessandro Lugli

**Affiliations:** 1 Translational Research Unit, Institute of Pathology, University of Bern, Murtenstrasse 31, Bern 3010, Switzerland; 2 Clinical Pathology Division, Institute of Pathology, University of Bern, Murtenstrasse 31, Bern 3010, Switzerland

**Keywords:** Tissue microarray, Next-generation, Tumor microenvironment, Automation, Pathology

## Abstract

**Background:**

Tissue microarray (TMA) technology revolutionized the investigation of potential biomarkers from paraffin-embedded tissues. However, conventional TMA construction is laborious, time-consuming and imprecise. Next-generation tissue microarrays (ngTMA) combine histological expertise with digital pathology and automated tissue microarraying. The aim of this study was to test the feasibility of ngTMA for the investigation of biomarkers within the tumor microenvironment (tumor center and invasion front) of six tumor types, using CD3, CD8 and CD45RO as an example.

**Methods:**

Ten cases each of malignant melanoma, lung, breast, gastric, prostate and colorectal cancers were reviewed. The most representative H&E slide was scanned and uploaded onto a digital slide management platform. Slides were viewed and seven TMA annotations of 1 mm in diameter were placed directly onto the digital slide. Different colors were used to identify the exact regions in normal tissue (n = 1), tumor center (n = 2), tumor front (n = 2), and tumor microenvironment at invasion front (n = 2) for subsequent punching. Donor blocks were loaded into an automated tissue microarrayer. Images of the donor block were superimposed with annotated digital slides. Exact annotated regions were punched out of each donor block and transferred into a TMA block. 420 tissue cores created two ngTMA blocks. H&E staining and immunohistochemistry for CD3, CD8 and CD45RO were performed.

**Results:**

All 60 slides were scanned automatically (total time < 10 hours), uploaded and viewed. Annotation time was 1 hour. The 60 donor blocks were loaded into the tissue microarrayer, simultaneously. Alignment of donor block images and digital slides was possible in less than 2 minutes/case. Automated punching of tissue cores and transfer took 12 seconds/core. Total ngTMA construction time was 1.4 hours. Stains for H&E and CD3, CD8 and CD45RO highlighted the precision with which ngTMA could capture regions of tumor-stroma interaction of each cancer and the T-lymphocytic immune reaction within the tumor microenvironment.

**Conclusion:**

Based on a manual selection criteria, ngTMA is able to precisely capture histological zones or cell types of interest in a precise and accurate way, aiding the pathological study of the tumor microenvironment. This approach would be advantageous for visualizing proteins, DNA, mRNA and microRNAs in specific cell types using *in situ* hybridization techniques.

## Introduction

In the late 1990s, tissue microarray (TMA) technology revolutionized the investigation of potential prognostic and predictive biomarkers [1]. TMAs involve the transfer of small tissue cores, typically 0.6 mm in diameter, from formalin-fixed paraffin-embedded (FFPE) tissues into an empty paraffin block [2]. This repeated transfer of tissue cores leads to the construction of a “tissue-archive” that can contain hundreds of tissue samples from a small or large number of patients. TMAs can be used to study tissue morphology, expression of proteins or genes and chromosomal aberrations using different stains, immunohistochemistry and *in situ* hybridization [3-5]. The combination of TMAs and clinically annotated samples represents an elegant and cost-effective approach to study panels of biomarkers under identical experimental conditions and to develop prognostic or predictive models of patient outcomes [6].

Despite these considerable advantages, conventional construction of TMAs is not without its limitations. TMA construction is often described as a meticulous, laborious and time-consuming process. A major drawback lies in the “approximation” of desired tissue spots for transfer into the TMA. Before tissues can be punched out of their blocks, the corresponding hematoxylin and eosin (H&E) slides are reviewed. The histological areas of interest are then marked using a felt-tip pen directly on the slide. When various tissue types are present, different colors and marking of assorted sizes are made. Pathologists familiar with TMA construction will confirm that annotating “specific” regions of interest in this manner is challenging. The manual alignment of the annotated region from an H&E slide to the donating block is not always exact.

It is now recognized that tumor heterogeneity can have a significant impact on the interpretation of biomarkers [7]. Increasingly important is the targeting of particular cell types, tissue types or histological zones using various core diameters of 0.6, 1.0, 1.5 or 2.0 mm. Traditional methods of tissue microarraying are suboptimal to achieve this goal; there is hence an urgent need to improve TMA technology to acquire histological precision, enhance the accuracy of punching and speed of construction. We have used the term next-generation TMAs (ngTMAs) to define the combination of cutting-edge digital pathology, automated tissue microarraying and histopathological expertise. The goal is to create TMAs containing the accurately determined histological structures with the aim of further optimizing biomarker research.

An example where ngTMAs would be useful is the investigation of the immune infiltrates in patients with different tumor types [8]. In colorectal cancers, immunohistochemical panels of immune infiltrates such as CD3, CD8 and CD45RO have been investigated in the tumor microenvironment within the main tumor body as well as at the invasion front.

The aim of this study was to determine the feasibility of ngTMAs to investigate biomarkers at the tumor-stroma interface within the main tumor body and invasion front of six tumor types: malignant melanomas, as well as lung, prostate, gastric, colon and breast cancers, using T-cell markers CD3, CD8 and CD45RO as an example.

## Materials and methods

### Case selection

In order to investigate the feasibility of ngTMAs for the assessment of host-related factors of the tumor microenvironment, six of the most frequently diagnosed tumor types were selected. Sixty cases were retrieved from the archives of the Institute of Pathology, University of Bern from 2009–2011 including 10 cases each of lung cancer, breast cancer, prostate cancer, gastric cancer, malignant melanoma and colon cancer. The diagnostic H&E slides were reviewed and the most representative one concerning the tumor microenvironment was selected for subsequent scanning. The use of patient material has been approved by the ethics committee of the Bern University Hospital (16-03-12).

### Slide scanning and annotation

The selected H&E slide of each whole tissue section was placed into a scanning cartridge and loaded into the slide scanner (Pannoramic P250, 3DHistech). All 60 slides could be processed in a single run. Digital images were uploaded onto the digital slide management platform Case Center (http://ngtma.path.unibe.ch/casecenter). Each slide could then be opened and viewed using Pannoramic Viewer software (3DHistech).

**Figure 1:**
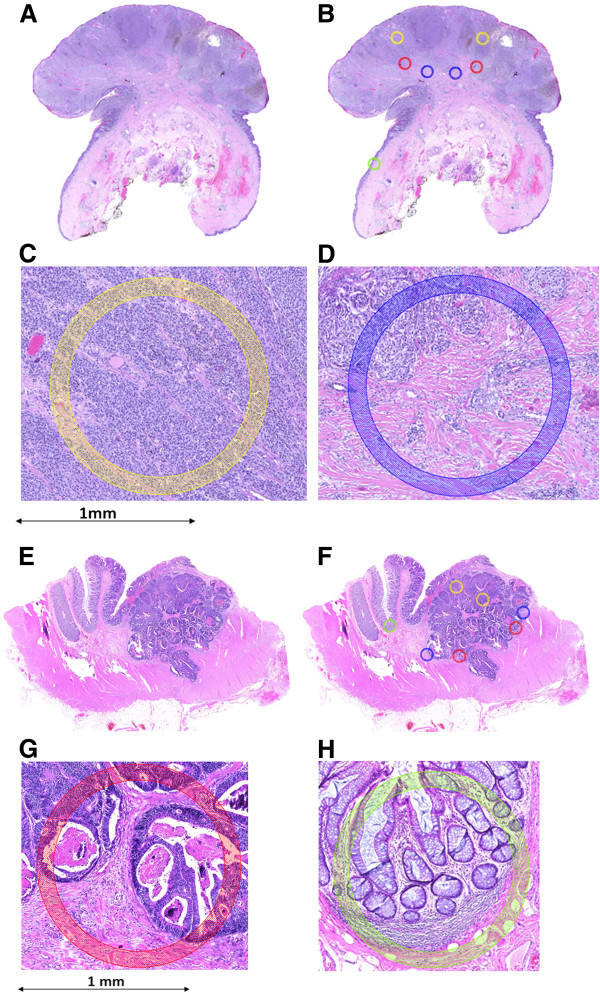
**Two examples showing scanned H&E sections, followed by annotations of specific histological regions and high-power magnifications of two different annotated regions. A**-**D**: malignant melanoma; **E**-**H**: colorectal cancer. Yellow annotation: tumor center, blue: tumor microenvironment at most invasive tumor front, red: invasion front, green: normal tissue. 5× magnification.

Using the 1.0 mm annotation tool, annotations of different colors corresponding to various histological areas were placed onto each digital slide. Although other sizes were available (0.6, 1.5 and 2.0 mm diameter annotation tools), we opted for 1.0 mm annotations for several reasons. Firstly, we wanted to capture large enough regions containing both tumor and stroma. Secondly, because of the potential heterogeneity of the tumor sample, several different tissue spots would need to be included. Thirdly, at least 1 normal tissue core per case would be necessary to provide a “control” for comparing staining patterns in tumors for different proteins or genes via immunostaining and fourthly, using 1 mm cores still leads to a minimum number of ngTMAs to be constructed. One green annotation was placed on normal tissue, two yellow annotations on the tumor center, two red annotations for the tumor periphery or invasion front and two blue annotations were used to capture areas of tumor budding and surrounding stroma (i.e., tumor microenvironment at the invasion front) (Figure 1). In total, 7 tissue spots were annotated per case, totaling 420 spots for subsequent punching and tissue microarraying.

### Automated tissue microarraying

The 60 donor blocks corresponding to the 60 annotated digital slides were loaded into the automated tissue microarrayer (Grandmaster, 3DHistech) and an image of each block was taken. Two empty paraffin embedded recipient blocks were simultaneously loaded. A tissue microarray layout was created to design a punching-schema for the final tissue microarray block. The created layout consisted of 243 tissue punches at 1 mm in diameter, suggesting that 2 ngTMA blocks would be sufficient for this study.

With the Grandmaster software, each digital slide could be retrieved from the Case Center. Using tissue landmarks, the image of the donor block and the digital slide were superimposed for exact correspondence. Annotations could then be viewed and confirmed. Punching of the donor blocks and transfer into the recipient block could be made after confirmation of each annotation and occurred in the sequence arranged with the layout. The result was 2 ngTMAs, the first containing tissue cores of lung, stomach, colon and breast and the second containing additional spots of breast, malignant melanoma and prostate tissues.

### Immunohistochemistry

The two ngTMAs were sectioned at 4 μm. After de-paraffination and rehydration, standard H&E staining was undertaken. In order to visualize the host-related immune markers, immunohistochemistry for the following antibodies was performed using an automated immunostainer (Leica, Bond III): Anti-CD3 (Abcam, clone SP7, tris buffer, 95° 30 minutes, 1:400), anti-CD8 (Dako, clone C8/144B, Tris buffer, 95° 20 minutes, 1:100) and anti-CD45RO (Abcam, clone UCH-L1, citrate buffer, 100° 30 minutes, 1:4000).

## Results

### Slide scanning and annotation

All 60 slides could be loaded and scanned automatically overnight. The time required for each scan varied in accordance with the size of the whole tissue section and ranged from 5 to 12 minutes. Total scanning time was approximately 10 hours. Slides were directly uploaded to the correct folder in the Case Center. Digital slide annotation could be easily and rapidly performed in 1 minute, on average. Therefore, 7 annotations on 60 cases could be performed within 1 hour.

### Automated tissue microarraying

The maximum capacity of the Grandmaster arrayer is 60 donor blocks therefore all selected blocks could be uploaded simultaneously. Land marking of donor block images and correspondence with digital slides was possible in less than 2 minutes per case followed by confirmation of annotated regions. Automated punching of tissue cores and transfer to the recipient block took 12 second per core. Punching could either be performed after confirming all annotations or after each single confirmed annotation. Total time for creating the 2 ngTMAs containing 420 cores at 1 mm in diameter was 1.4 hours.

Less than 2% of all tissue cores were unsuccessfully transferred from the donor block to the recipient block. These cores could however be recuperated in a second attempt resulting in a 100% successful transfer rate.

### The tumor microenvironment is captured using ngTMA

**Figure 2:**
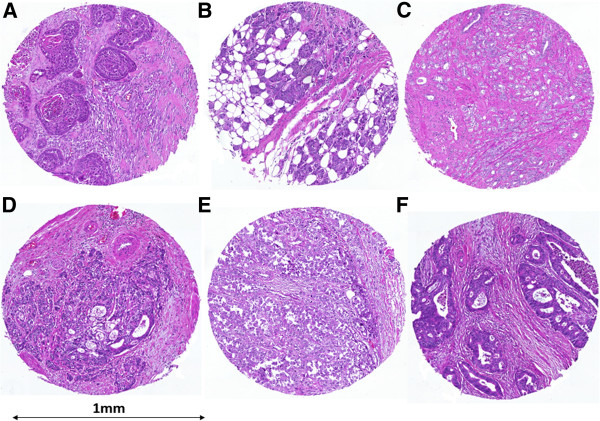
**Representative H&E stains of ngTMA tissue spots. A**) lung cancer, **B**) breast cancer, **C**) prostate cancer, **D**) gastric cancer, **E**) malignant melanoma and **F**) colorectal cancer; 10× magnification.

In Figure 2, representative images of H&E stained spots from the two ngTMAs containing each of the 6 tissue types of interest are shown. These outline the precision with which ngTMA could capture regions of tumor-stroma interaction at the invasion front of each cancer.

### Evaluation of the tumor microenvironment: host-related features

**Figure 3:**
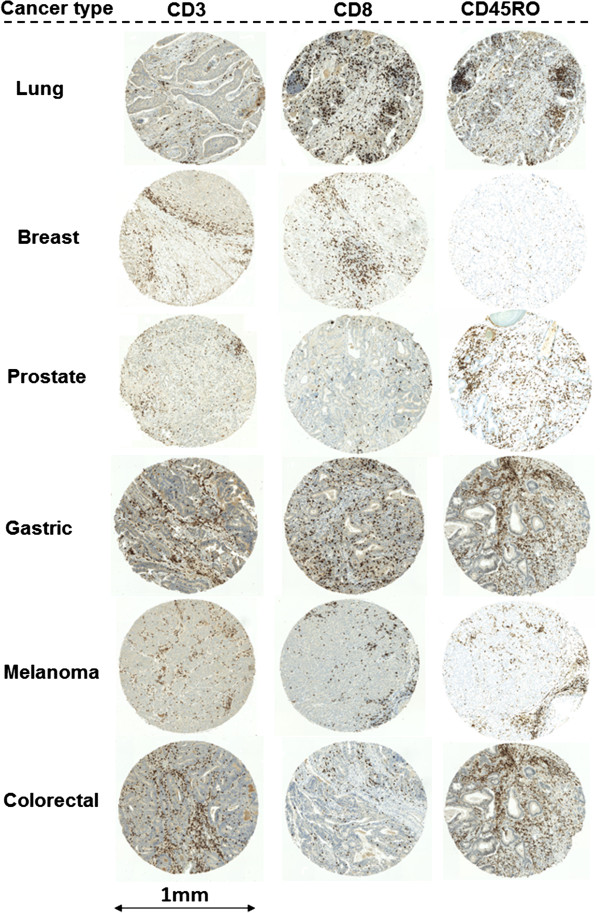
**Representative immunohistochemistry staining for CD3, CD8 and CD45RO and each cancer type; 5× magnification.**

Immunohistochemistry for anti-CD3, anti-CD8 and anti-CD45RO highlights the T-lymphocytic immune reaction in the microenvironment of the tumor within the tumor center or invasion front across each tumor type (Figure 3).

## Discussion

**Figure 4:**
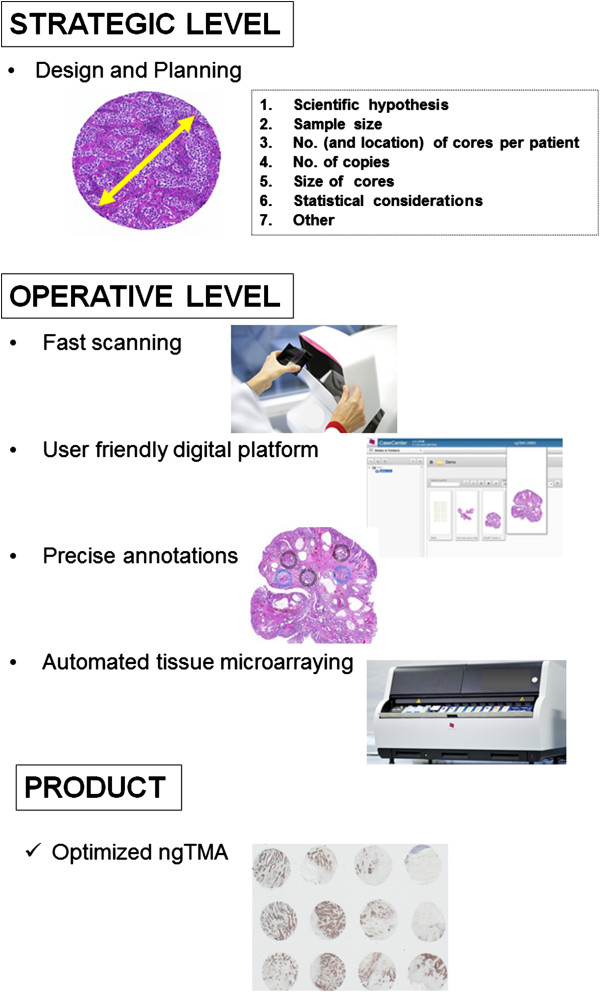
**The ngTMA process: strategic and operative levels.** The first step in the construction of ngTMAs is the design and planning in terms of the scientific hypothesis, available material, case selection, as well as TMA and statistical considerations. The operative level begins by slide scanning and the use of a digital platform. Histological expertise is necessary to perform precise annotations. Automated tissue microarraying ensues. The final product is an optimized ngTMA which will allow the user to answer the hypothesis in question.

The UICC/AJCC TNM classification is still regarded as the gold standard for prognostication in colorectal cancer. Nevertheless, TNM staging is recognized as imperfect and is continuously updated. Consequently, the need for new biomarkers is urgent. This has led to an inundation of studies proposing novel and promising prognostic factors. The lack of reproducibility and standardization often leads to a rejection of such biomarkers, perhaps prematurely in some cases. Over the last 15 years, TMA technology has played a major role in the translation of biomarkers into daily diagnostic practice, but its use is often criticized as laborious, imprecise and time-consuming; additionally, improper planning on the strategic level decreases the strength of TMA investigations further. Therefore, the simple implementation of an automated TMA instrument cannot be the definition of ngTMA. The construction of ngTMA is a well-defined process including the following steps (Figure 4):

### Planning and design

Based on scientific hypothesis and requirements, an interdisciplinary research task force should consider statistical issues such as sample size and the number of tissue punches per patient depending on the tumor types and especially from which tumor location. This step should be considered the “strategic level” of ngTMA and should not be under-estimated to avoid waste of time, material, human and financial resources; *fast scanning and user friendly digital platform*: these steps allow the visualization of slides using a digital platform which is the interface between all research groups involved and allows the discussion of cases without the need for additional meetings that are often logistically difficult to organize; *precise annotations*: this step is likely the most crucial as it requires experience in morphology to exactly determine the tumor locations from which the tissue punches should be taken from. In colorectal cancer, for example, a well-constructed ngTMA should include punches from normal tissue, tumor center, tumor front, intra-tumoral and peri-tumoral microenvironment and if available preoperative biopsies, lymph nodes or distant metastases. Differentiating these various histological “zones” may be challenging to the untrained eye and hence, an optimal ngTMA would be constructed in collaboration with pathologists; *automated punching*: this step facilitates the working process by its precision and flexibility; the choice of the core size depends on the scientific hypothesis; more “functional” ngTMAs such as one investigating epithelial-mesenchymal transition (EMT) may require a smaller sample size but larger tissue cores areas (1.5-2.0 mm in diameter), whereas more high-throughput studies could be performed using the “traditional” 0.6 mm punch size. In our experience using a conventional arrayer, an average of 15 cores can be punched and transferred per hour. Donor block punching and transfer to the recipient block took 12 seconds per core, thus significantly reducing the time and human resources necessary for TMA construction.

In the present analysis we tested whether the new approach of “ngTMA” could be an ideal platform to study the tumor microenvironment of different solid tumors. Indeed, the selected immune markers CD3, CD8 and CD45RO which are proposed as important prognostic immune infiltrates in the literature [8] could be visualized by immunohistochemistry in precisely targeted tissue punches from the microenvironment of the tumor center and the tumor invasive front. This approach may even become more important in the future when not only proteins, but also mRNA and microRNA can be visualized on paraffin embedded tissues using *in situ* hybridization techniques.

## Competing interests

The authors declare that they have no competing interests.

## Authors’ contributions

IZ was involved in the conception of the study, performed the TMA construction and technical work; VK and HD performed the case selection, review and slide annotation; AP participated in conception and study design; AL made substantial contributions to conception and study design in addition to case review and slide annotation. All authors read and approved the final manuscript.

## References

[B1] Kallioniemi OP Wagner U Kononen J Sauter G Tissue microarray technology for high-throughput molecular profiling of cancer Hum Mol Genet 2001 10 657 10.1093/hmg/10.7.657 11257096

[B2] Voduc D Kenney C Nielsen TO Tissue microarrays in clinical oncology Semin Radiat Oncol 2008 18 89 10.1016/j.semradonc.2007.10.006 18314063 PMC2292098

[B3] Fleischmann A Rotzer D Seiler R Studer UE Thalmann GN Her2 amplification is significantly more frequent in lymph node metastases from urothelial bladder cancer than in the primary tumours Eur Urol 2011 60 350 10.1016/j.eururo.2011.05.035 21640482

[B4] Tapia C HER2 analysis in breast cancer: reduced immunoreactivity in FISH non-informative cancer biopsies Int J Oncol 2004 25 1551 15547690

[B5] Zlobec I Node-negative colorectal cancer at high risk of distant metastasis identified by combined analysis of lymph node status, vascular invasion, and Raf-1 kinase inhibitor protein expression Clin Cancer Res 2008 14 143 10.1158/1078-0432.CCR-07-1380 18172264

[B6] Zlobec I Role of RHAMM within the hierarchy of well-established prognostic factors in colorectal cancer Gut 2008 57 1413 10.1136/gut.2007.141192 18436576

[B7] Rehemtulla A Overcoming intratumor heterogeneity of polygenic cancer drug resistance with improved biomarker integration Neoplasia 2012 14 1278 23308059 10.1593/neo.122096 PMC3540957

[B8] Galon J Cancer classification using the Immunoscore: a worldwide task force J Transl Med 2012 10 205 10.1186/1479-5876-10-205 23034130 PMC3554496

